# Development and evaluation of a case-based learning program integrated with the BOPPPS model in neurosurgical residency training: a prospective controlled educational study

**DOI:** 10.3389/fmed.2026.1929057

**Published:** 2026-08-28

**Authors:** Jun Shen, Zhengyang Zhang, Qi Zhang, Zihao Zhou, Ziji Li, Shaolin Zhang, Qian An, Pengcheng Xu

**Affiliations:** 1 Department of Neurosurgery, The First Affiliated Hospital of Wannan Medical University (YiJiShan Hospital of Wannan Medical University), Wuhu, Anhui, China; 2 Department of Respiratory and Critical Care Medicine, Wuhu Hospital of Traditional Chinese Medicine, Wuhu, Anhui, China

**Keywords:** BOPPPS, Case-based learning, Clinical competency, competency-based medical education, Kirkpatrick model, neurosurgical residency training, Objective Structured clinical examination

## Abstract

**Background:**

Neurosurgical residency training requires teaching strategies that promote clinical reasoning and competency development beyond traditional lecture-based instruction. Although case-based learning (CBL) and the BOPPPS instructional model have each shown educational benefits, evidence regarding their integrated application in neurosurgical residency training remains limited. This study aimed to develop and evaluate a structured CBL program integrated with the BOPPPS instructional model and to assess its educational impact within the context of neurosurgical residency training.

**Methods:**

This study developed and implemented a CBL program integrated with the BOPPPS instructional model for neurosurgical residents. Participants were assigned to either a CBL-BOPPPS group or a traditional lecture-based teaching group for theoretical instruction. Following classroom teaching, both groups received identical ward-based clinical teaching involving real patients. Educational outcomes were evaluated using a multidimensional framework based on the Kirkpatrick model, including learner satisfaction, written examinations, Objective Structured Clinical Examination (OSCE) performance, self-perceived clinical competence, and teaching workload. Between-group comparisons were conducted using appropriate statistical analyses.

**Results:**

Compared to traditional teaching methods, the CBL-BOPPPS group exhibited significantly higher learner satisfaction and superior performance in higher-order knowledge application, clinical reasoning, treatment planning, and communication skills. Additionally, OSCE-based clinical performance and self-perceived clinical competence showed significant improvement. However, no significant differences were observed between the groups in foundational knowledge acquisition or imaging-based interpretation. Faculty preparation time and perceived teaching workload were greater in the CBL-BOPPPS group.

**Conclusion:**

The CBL-BOPPPS model represents a practical and educationally valuable approach to neurosurgical residency training. Its benefits appear to be domain-specific, preferentially enhancing higher-order competencies aligned with competency-based education while maintaining the role of traditional lectures for efficient foundational knowledge transmission. These findings support a blended instructional strategy in postgraduate neurosurgical education.

## 1 Introduction

Neurosurgical residency training is one of the most demanding forms of postgraduate medical education, requiring residents to integrate extensive theoretical knowledge, clinical reasoning, and advanced procedural skills while making high-stakes clinical decisions. Traditionally, residency training has relied on an apprenticeship model centered on operating room experience, bedside teaching, and graduated clinical responsibility (1, 2). Although this approach remains fundamental, there is increasing emphasis on competency-based medical education (CBME), which focuses on clearly defined learning outcomes, deliberate practice, and objective assessment of clinical competence rather than time-based training alone (3, 4). Achieving these educational goals requires instructional strategies that actively engage residents in clinical reasoning and decision-making, rather than relying solely on passive knowledge acquisition.

Case-based learning (CBL) has emerged as a learner-centered instructional approach that enhances clinical reasoning, problem-solving skills, and long-term knowledge retention by situating learning within authentic clinical contexts (5). Previous studies have demonstrated that CBL increases learner engagement and perceived clinical relevance in undergraduate neurosurgical education (6, 7). However, the educational demands of residency training differ significantly from those of undergraduate education, as residents are expected to apply theoretical knowledge to real-time clinical decision-making and patient management. Moreover, most existing CBL studies in neurosurgery focus on undergraduate learners, lack standardized instructional frameworks, and offer limited guidance for reproducible implementation in residency education (8).

The BOPPPS instructional model (Bridge-in, Objective, Pre-assessment, Participatory Learning, Post-assessment, and Summary) provides a structured framework that promotes learner engagement, formative assessment, and constructive alignment among learning objectives, teaching activities, and educational outcomes (9, 10). Integrating CBL with the BOPPPS model may enhance the consistency and effectiveness of case-based instruction while supporting the competency-oriented goals of competency-based medical education (CBME). However, evidence regarding the application of this integrated teaching strategy in neurosurgical residency training remains limited. Furthermore, few studies have utilized comprehensive evaluation frameworks, such as the Kirkpatrick model, to assess educational outcomes across multiple dimensions, including learner satisfaction, knowledge acquisition, behavioral performance, and educational impact (11).

To address these gaps, we developed a standardized CBL program integrated with the BOPPPS instructional model, specifically designed for neurosurgical residency training. Guided by CBME principles and evaluated using the Kirkpatrick framework. This prospective controlled study aimed to determine whether the CBL-BOPPPS approach could improve learner satisfaction, promote higher-order knowledge application, improve clinical performance, and increase self-perceived competence compared to traditional lecture-based teaching.

## 2 Methods

### 2.1 Study design

**Figure 1:**
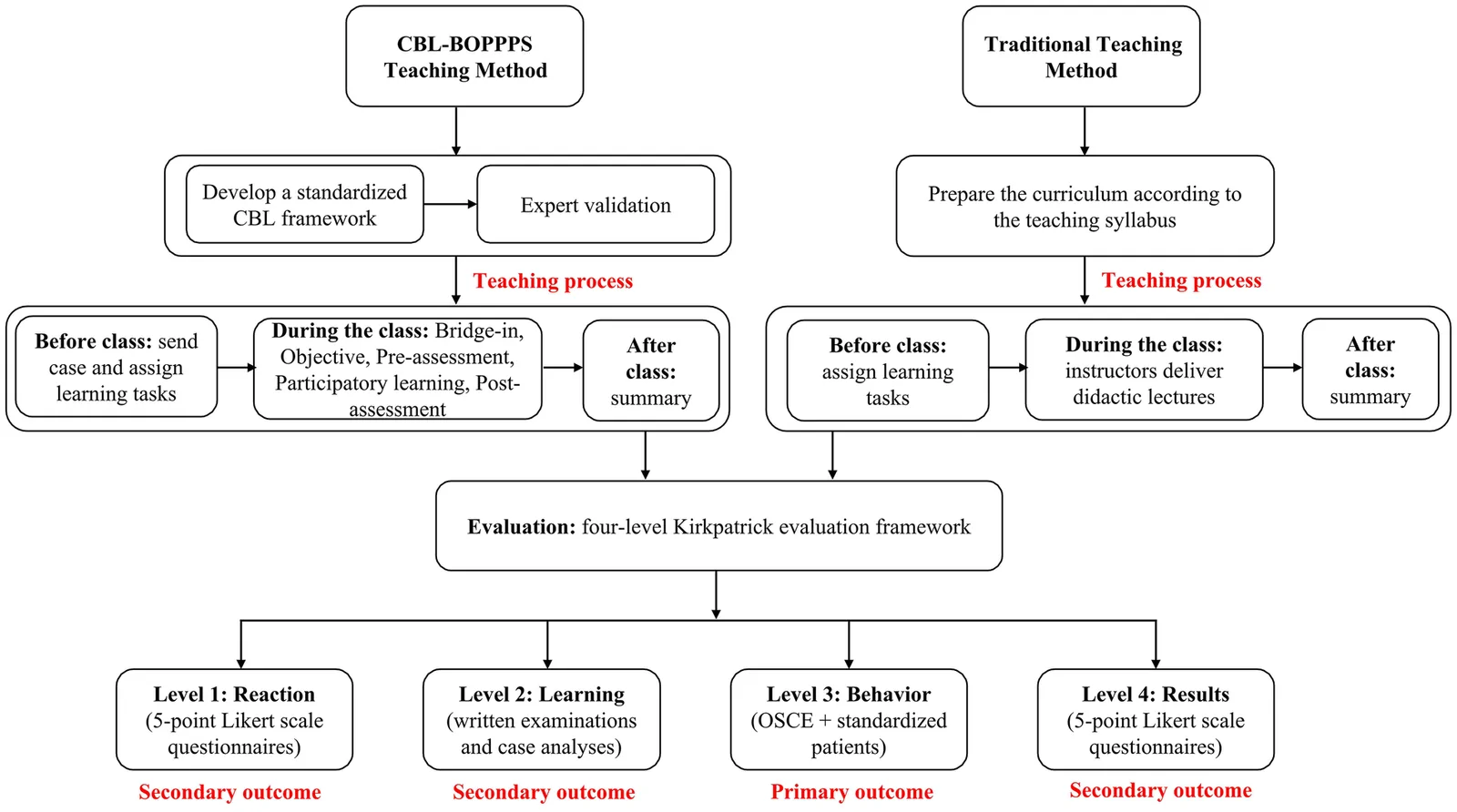
Flowchart of the Study.

The flowchart for this study is shown in Figure 1. This study employed a prospective, controlled educational intervention design to evaluate the effectiveness of a CBL program integrated with the BOPPPS instructional model in neurosurgical residency training. The study was conducted within the standardized neurosurgery residency program at the First Affiliated Hospital of Wannan Medical University (YiJiShan Hospital of Wannan Medical University). To minimize contamination between teaching methods, cluster randomization based on predefined neurosurgical rotation blocks was adopted. Each rotation block was randomly assigned to either the CBL-BOPPPS intervention or the traditional lecture-based teaching group before the start of the rotation. The educational framework was grounded in adult learning theory, experiential learning, and CBME, emphasizing clinical reasoning, clinical decision-making, and the transfer of learning into real clinical practice (3, 12, 13). Educational outcomes were evaluated using the four-level Kirkpatrick model, assessing learners’ reactions, learning outcomes, behavioral performance, and perceived educational impact (11).

### 2.2 Participants

A consecutive sampling strategy was adopted. All residents who completed their neurosurgical rotation at the First Affiliated Hospital of Wannan Medical University between September 2023 and October 2025 and met the study eligibility criteria were invited to participate. Residents who declined participation or did not complete all study assessments were excluded. A total of 76 residents were enrolled. Participants were allocated according to predefined neurosurgical rotation blocks. Before each rotation period commenced, the rotation block was randomly assigned to either the CBL-BOPPPS group or the traditional teaching group using a computer-generated random sequence. Consequently, all residents within the same rotation block received the same instructional approach, thereby minimizing contamination between teaching methods. This cluster randomization strategy resulted in 38 residents in the CBL-BOPPPS group and 38 residents in the traditional teaching group. Baseline characteristics, including age, sex, residency training year, theoretical knowledge, technical skills, and clinical reasoning ability, were comparable between the two groups. Because this was a prospective educational intervention conducted at a single residency training center, the number of eligible residents was determined by the annual residency enrollment during the study period. Therefore, all eligible residents were consecutively included, and no formal *a priori* sample size calculation was performed, consistent with previous residency-based medical education studies (14, 15).

### 2.3 Curriculum design and teaching models

The curriculum focused on core neurosurgical diseases commonly encountered during residency training, with learning objectives aligned to diagnostic reasoning, operative decision-making, and perioperative management. Two teaching models were implemented: the CBL-BOPPPS group and the traditional teaching group. In the CBL-BOPPPS group, students participated in a structured case-based learning curriculum integrated with the BOPPPS model. In the traditional teaching group, students received conventional lecture-based instruction. Both groups received the same total instructional time and covered identical disease categories to ensure educational equivalence.

### 2.4 Development of the standardized CBL case framework

A standardized CBL framework was developed specifically for residency-level training, emphasizing higher-order clinical competencies beyond undergraduate education. Each case followed a unified structure, including the chief complaint and history of present illness, physical and neurological examination findings, imaging and auxiliary examinations, differential diagnosis, surgical indications and operative strategy, perioperative risk assessment, postoperative management, and complication prevention. The selected neurosurgical conditions included traumatic intracerebral hematoma, vestibular schwannoma, temporal lobe glioma, pituitary adenoma, spinal meningioma, and posterior communicating artery aneurysm. These cases were chosen to reflect progressive complexity, encouraging students to integrate theoretical knowledge with real-world clinical decision-making (16).

A structured and standardized CBL framework tailored for neurosurgical residency training was developed through an iterative, expert-driven refinement process. An expert panel comprising four senior neurosurgeons with extensive experience in residency training, curriculum development, and clinical education was convened to guide case development. Initial case drafts were created based on real clinical scenarios encountered during routine neurosurgical practice. The expert panel reviewed each case with a focus on clinical authenticity, cognitive complexity, alignment with residency-level competencies, and feasibility for structured discussion. Feedback was collected through multiple rounds of focused group discussions, and cases were revised accordingly until consensus was reached regarding content completeness, instructional clarity, and educational relevance. Compared to undergraduate CBL cases, the residency-level cases placed greater emphasis on diagnostic uncertainty, operative strategy selection, perioperative risk assessment, and postoperative decision-making, ensuring alignment with competency-based residency training objectives.

### 2.5 Teaching process

#### 2.5.1 CBL-BOPPPS teaching model

The CBL-BOPPPS teaching model integrates case-based learning with the BOPPPS instructional framework and is implemented in a structured, stepwise manner that emphasizes active learning, clinical reasoning, and competency development (17, 18).

**Before class**, faculty members selected representative neurosurgical cases aligned with predefined learning objectives and resident training requirements. Each case included standardized clinical materials, such as patient history, imaging data, laboratory results, and key clinical decision points. These materials were distributed to residents via the WeChat group prior to class. Residents were organized into small groups and instructed to independently review the cases, consult relevant textbooks and clinical guidelines, and identify potential diagnostic and therapeutic strategies. This phase corresponded to the pre-assessment component of the BOPPPS model, allowing learners to activate prior knowledge and identify learning gaps.

**During the class**, teaching followed the complete BOPPPS sequence. The session began with a Bridge-in, during which instructors introduced a clinical scenario to simulate a real-world neurosurgical context and stimulate learner engagement. Clear learning objectives were then explicitly stated, outlining the expected competencies in diagnosis, decision-making, and patient management. In the Participatory learning phase, residents engaged in guided group discussions, analyzing the case step by step, including interpretation of clinical data, radiological findings, differential diagnosis, selection of surgical or conservative treatment strategies, and perioperative management considerations. Faculty members acted primarily as facilitators rather than lecturers, prompting critical thinking, challenging assumptions, and encouraging evidence-based reasoning. Formative post-assessment was conducted through targeted questioning, case-based quizzes, or brief group presentations to assess residents’ understanding and clinical reasoning processes.

**After class**, instructors summarized consolidated key knowledge points, highlighted common pitfalls, and linked theoretical concepts to clinical practice.

#### 2.5.2 Traditional teaching model

The traditional teaching model employed a lecture-centered instructional approach supplemented by routine clinical observation.

**Before class**, faculty members developed structured teaching outlines based on the standard neurosurgery curriculum and recommended textbooks. These materials were distributed to residents in advance via the class WeChat group. Trainees were required to independently review the assigned textbook content and familiarize themselves with the basic theoretical framework of each disease prior to the scheduled lecture.

**During class**, instructors delivered didactic lectures focusing on core theoretical components, including disease definitions, underlying pathophysiology, clinical presentations, imaging characteristics, diagnostic criteria, and guideline-based treatment strategies. Teaching was predominantly instructor-driven, with residents primarily receiving information through listening and note-taking. Classroom interaction was limited and generally consisted of brief questions posed by instructors to reinforce key concepts.

**After class,** instructors provided a concise summary of the lecture content, emphasizing essential knowledge points and examination-relevant concepts.

It is important to emphasize that the intervention focused exclusively on the theoretical teaching component. After completing classroom-based instruction, students in both groups participated in identical ward-based clinical teaching, during which supervising faculty provided bedside instruction using real patients, including history taking, physical examination, and clinical explanation. This design ensured that differences in outcomes could be primarily attributed to the instructional approach used during the theoretical teaching phase rather than to disparities in clinical exposure.

### 2.6 Evaluation system design based on the Kirkpatrick model

An outcome evaluation framework based on the four-level Kirkpatrick model was employed to comprehensively assess the effectiveness of the CBL-BOPPPS intervention in neurosurgical residency training. This framework encompassed learners’ reactions, learning gains, behavioral performance, and educational impact.

#### Level 1: reaction

Students’ immediate perceptions of the teaching intervention were evaluated using a structured satisfaction questionnaire. This instrument assessed multiple dimensions, including learning engagement, clarity of instruction, alignment with clinical practice, and overall satisfaction with the teaching approach. All items were rated on a 5-point Likert scale, ranging from 1 (strongly disagree) to 5 (strongly agree).

#### Level 2: learning

Learning outcomes were assessed through a combination of written knowledge examinations and structured clinical case analyses. The case analysis component emphasized higher-level cognitive skills essential for residency training, including clinical data interpretation, radiological image interpretation, diagnosis and differential diagnosis, selection of the appropriate surgical plan, and postoperative management. Performance scores were quantitatively compared between groups.

#### Level 3: behavior

Clinical performance and observable behavioral outcomes were assessed using an Objective Structured Clinical Examination (OSCE) with standardized patients. The OSCE stations were designed to simulate authentic neurosurgical scenarios and evaluated students’ competencies in medical history collection, physical examination, formulating a comprehensive plan for additional imaging and laboratory examinations, imaging interpretation, diagnostic accuracy, treatment completeness, and patient communication skills. The OSCE was independently evaluated by two neurosurgeons (attending physician level or above) with extensive experience in neurosurgical education. Before the assessments, both evaluators received standardized training regarding the scoring criteria and assessment procedures to ensure consistency. Evaluators were blinded to the participants’ group allocation throughout the assessment process. Standardized scoring rubrics were used for all stations. The final score for each station was calculated as the average of the two examiners’ scores.

#### Level 4: results

The educational impact at the results level was evaluated through students’ self-perceived competence. This assessment included their confidence in clinical decision-making, ability to manage complex cases, stress management in clinical settings, clinical thinking skills, communication abilities, and teamwork capacity. Responses were recorded using a 5-point Likert scale. The student and teacher questionnaires were adapted from our previously published questionnaires developed for neurosurgical education (6, 7). Minor modifications were made to reflect the objectives of the present study while maintaining the original questionnaire structure and core evaluation domains. The questionnaires consisted of Likert-scale items assessing theoretical knowledge, self-directed learning, learning interest, learning motivation, teamwork, clinical reasoning, communication skills, learning burden, teaching burden, teaching sustainability, and overall satisfaction. Before implementation, the revised questionnaires were reviewed by the departmental teaching committee to ensure content relevance and clarity.

#### Measures

Outcome variables were predefined in accordance with the Kirkpatrick Evaluation Framework. The primary outcome was the residents’ performance on the OSCE, which included medical history collection, physical examination, formulate a comprehensive plan for additional imaging and laboratory examinations, imaging interpretation, diagnostic accuracy, treatment completeness, and patient communication skills. Secondary outcomes included learner satisfaction, theoretical examination scores, questionnaire-based self-perceived competence, and teacher evaluations.

### 2.7 Statistical analysis

Statistical analyses were performed using SPSS software (version 22.0; IBM Corp., Armonk, NY, USA). Continuous variables are presented as mean ± standard deviation (SD), whereas categorical variables are presented as frequencies and percentages. Before analysis, the normality of continuous variables was assessed using the Shapiro–Wilk test. Variables with an approximately normal distribution were compared using the independent-samples *t* test, whereas non-normally distributed variables were analyzed using the Mann–Whitney *U* test. Categorical variables were compared using the chi-square test or Fisher's exact test, as appropriate. All statistical tests were two-sided, and a *P* value < 0.05 was considered statistically significant.

## 3 Results

### 3.1 Baseline characteristics

A total of 76 neurosurgical residents were enrolled and evenly assigned to the CBL-BOPPPS group (*n* = 38) and the traditional teaching group (*n* = 38). As shown in Table 1, no statistically significant differences were observed between the two groups regarding age, sex distribution, or residency training grade (all *P* > 0.05). Baseline assessments of theoretical knowledge, technical skills, and clinical reasoning scores were also comparable between groups, indicating adequate baseline equivalence prior to the intervention.

**Table 1:** Comparison of baseline characteristics between two groups.

Characteristic	CBL-BOPPPS Group (*n* = 38)	Traditional Group (*n* = 38)	*P* value
Age (years)	25.6 ± 1.8	26.0 ± 1.9	0.292
Sex			1.000
Male (*n*, %)	35 (92.1%)	36 (94.7%)	
Female (*n*, %)	3 (7.9%)	2 (5.3%)	
Neurosurgical residency training grade			0.830
The first year (*n*, %)	23 (60.5%)	24 (63.2%)	
The second year (*n*, %)	7 (18.4%)	8 (21.0%)	
The third year (*n*, %)	8 (21.1%)	6 (15.8%)	
Baseline theoretical knowledge score (point)	80.2 ± 7.5	81.3 ± 7.8	0.550
Baseline technical skills score (point)	79.2 ± 8.7	81.6 ± 7.5	0.193
Clinical reasoning score (point)	75.5 ± 8.9	75.1 ± 8.4	0.853

The maximum score for baseline theoretical knowledge, baseline technical skills, and clinical reasoning is 100 points.

### 3.2 Learner satisfaction (Kirkpatrick level 1: reaction)

Learner satisfaction outcomes are summarized in Table 2. Residents in the CBL-BOPPPS group reported significantly higher levels of satisfaction across multiple dimensions compared to those in the traditional group. Specifically, the CBL-BOPPPS group demonstrated greater perceived enhancement of active participation (*P* = 0.013), encouragement of independent thinking (*P* = 0.044), clarity of learning objectives (*P* = 0.040), and clarity of key concepts (*P* = 0.025). Additionally, residents receiving CBL-BOPPPS instruction reported stronger perceptions of theory–practice integration (*P* = 0.042) and higher overall satisfaction with the teaching method (*P* = 0.025). Willingness to recommend the teaching approach to peers was also significantly higher in the CBL-BOPPPS group (*P* < 0.001).

**Table 2:** Comparison of learner satisfaction between CBL-BOPPPS group and traditional group (Kirkpatrick level 1: reaction).

Items	CBL-BOPPPS Group (*n* = 38)	Traditional Group (*n* = 38)	*P* value
The teaching method enhanced my active participation in learning	4.4 ± 0.8	3.9 ± 1.0	0.013
The course encouraged me to think independently	4.5 ± 0.7	4.1 ± 0.9	0.044
Learning objectives were clearly explained	4.5 ± 0.8	4.0 ± 1.0	0.040
Key concepts were presented in a clear and understandable manner	4.5 ± 0.7	4.1 ± 1.0	0.025
The teaching helped bridge theory and clinical application	4.5 ± 0.7	4.2 ± 0.9	0.042
Overall satisfaction with the teaching method	4.6 ± 0.7	4.1 ± 1.0	0.025
I would recommend this teaching method to other students	4.7 ± 0.6	4.1 ± 0.8	< 0.001

The maximum score for each item is 5 points.

### 3.3 Knowledge acquisition (Kirkpatrick level 2: learning)

Comparisons of knowledge acquisition outcomes are presented in Table 3. No significant difference was observed between groups in written knowledge test scores (*P* = 0.127). However, the CBL-BOPPPS group achieved a significantly higher total score in clinical case analysis (*P* = 0.003). Subdomain analysis revealed that residents in the CBL-BOPPPS group performed significantly better in diagnosis and differential diagnosis (*P* = 0.037), selection of appropriate surgical plans (*P* = 0.030), and postoperative management (*P* = 0.036). No significant differences were found between groups in clinical data interpretation or radiological image interpretation (both *P* > 0.05).

**Table 3:** Comparison of knowledge acquisition between CBL group and traditional group (kirkpatrick level 2: learning).

Items	CBL-BOPPPS Group (*n* = 38)	Traditional Group (*n* = 38)	*P* value
Written knowledge test score (0–100)	82.9 ± 7.5	80.2 ± 7.7	0.127
Clinical case analysis total score (0–100)	73.8 ± 6.4	69.4 ± 5.7	0.003
Clinical data interpretation (0–20)	15.7 ± 2.3	15.9 ± 2.1	0.644
Radiological image interpretation (0–20)	15.5 ± 2.4	15.3 ± 2.5	0.641
Diagnosis and differential diagnosis (0–20)	15.2 ± 2.4	14.0 ± 2.8	0.037
Select the appropriate surgical plan (0–20)	14.4 ± 2.6	12.9 ± 3.3	0.030
Postoperative management (0–20)	12.9 ± 2.7	11.4 ± 3.5	0.036

The maximum score for written knowledge and clinical case analysis is 100 points each. The maximum score for each item in the clinical case analysis is 20 points.

### 3.4 Clinical performance and behavioral outcomes (Kirkpatrick level 3: behavior)

OSCE-based assessments of clinical performance and behavioral outcomes are detailed in Table 4. The CBL-BOPPPS group demonstrated significantly higher scores in medical history collection (*P* = 0.013), physical examination (*P* = 0.019), formulation of comprehensive plans for additional imaging and laboratory examinations (*P* = 0.037), and patient communication skills (*P* = 0.030). No statistically significant differences were observed in imaging interpretation, diagnostic accuracy, or treatment completeness between the two groups. Importantly, the overall OSCE score was significantly higher in the CBL-BOPPPS group compared to the traditional group (*P* = 0.001).

**Table 4:** Comparison of OSCE-based clinical performance and behavioral outcomes between CBL group and traditional group (kirkpatrick level 3: behavior).

Items	CBL-BOPPPS Group (*n* = 38)	Traditional Group (*n* = 38)	*P* value
Medical history collection (0–15)	11.2 ± 2.0	10.1 ± 2.3	0.013
Physical examination (0–15)	9.9 ± 2.1	8.7 ± 2.5	0.019
Formulate a comprehensive plan for additional imaging and laboratory examinations (0–15)	12.2 ± 1.7	11.3 ± 2.0	0.037
Imaging interpretation (0–15)	11.6 ± 1.9	11.6 ± 2.2	0.955
Diagnostic accuracy (0–15)	11.6 ± 2.0	11.4 ± 2.3	0.709
Treatment completeness (0–15)	11.2 ± 1.9	10.7 ± 2.2	0.300
Skills in patient communication (0–10)	6.6 ± 1.7	5.6 ± 2.2	0.030
Overall OSCE score (0–100)	74.4 ± 5.7	69.3 ± 6.8	0.001

The maximum score for skills in patient communication is 10 points; for each of the other items, it is 20 points; and the overall OSCE score is 100 points.

### 3.5 Self-perceived competence (Kirkpatrick level 4: results)

Self-perceived competence outcomes are presented in Table 5. Residents in the CBL-BOPPPS group reported significantly higher scores in learning motivation (*P* = 0.021), targeted and engaging learning experiences (*P* = 0.037), patient management (*P* = 0.046), management of complex cases (*P* = 0.048), stress management in clinical settings (*P* = 0.041), clinical thinking ability (*P* = 0.043), clinician–patient communication skills (*P* = 0.038), and teamwork skills (*P* = 0.016). No significant differences were observed between groups in self-learning skills or basic knowledge mastery (both *P* > 0.05).

**Table 5:** Comparison of self-perceived competence between CBL group and traditional group (Kirkpatrick level 4: results).

Items	CBL-BOPPPS Group (*n* = 38)	Traditional Group (*n* = 38)	*P* value
Learning motivation (point)	4.4 ± 0.8	4.0 ± 1.0	0.021
Self-learning skills (point)	4.4 ± 0.7	4.3 ± 0.8	0.638
Learning more targetedlly and more interestingly (point)	4.5 ± 0.7	4.1 ± 1.0	0.037
Basic knowledge mastery (point)	4.2 ± 0.9	4.2 ± 1.0	0.906
Patient management	4.1 ± 1.0	3.7 ± 1.0	0.046
Manage complex cases	4.1 ± 1.0	3.6 ± 1.1	0.048
Manage stress experienced in the clinical setting	4.3 ± 1.0	3.8 ± 0.9	0.041
Clinical thinking ability (point)	4.4 ± 0.7	4.1 ± 0.8	0.043
Clinician-patient communication skills (point)	4.4 ± 0.8	3.9 ± 1.0	0.038
Teamwork skills (point)	4.4 ± 0.8	4.0 ± 0.9	0.016

The maximum score for each item is 5 points.

### 3.6 Teaching efficiency and educational burden

Teaching efficiency and educational burden are summarized in Table 6. Compared to the traditional teaching group, the CBL-BOPPPS group required significantly more preparation time per case (*P* < 0.001), experienced a higher perceived teaching workload (*P* < 0.001), and reported greater anxiety related to class preparation (*P* = 0.002). Despite these increases in workload-related factors, no significant difference was observed in perceived teaching sustainability between the groups (*P* = 0.491). Notably, overall teaching quality scores were significantly higher in the CBL-BOPPPS group than in the traditional group (*P* = 0.003).

**Table 6:** Teaching efficiency and educational burden between CBL group and traditional group.

Items	CBL-BOPPPS Group (*n* = 16)	Traditional Group (*n* = 13)	P value
Preparation time required for each case (hours)	4.1 ± 0.8	2.5 ± 0.6	< 0.001
Increase the workload for teachers	4.5 ± 0.6	3.3 ± 0.9	< 0.001
Anxiety about preparing for class	4.4 ± 0.7	3.3 ± 1.0	0.002
Teaching sustainability	4.4 ± 0.8	4.2 ± 0.9	0.491
Teaching quality score	86.1 ± 6.1	78.2 ± 6.9	0.003

The maximum score for teaching quality is 100 points, while the maximum score for each of the other items is 5 points (The number of participating teachers differed between groups because the CBL-BOPPPS approach employed small-group facilitation, requiring more instructors than the traditional lecture-based format).

## 4 Discussion

In this study, we developed and evaluated a CBL program integrated with the BOPPPS instructional model within the context of neurosurgical residency training. Using a multidimensional evaluation framework based on the Kirkpatrick model, the findings suggest that the CBL-BOPPPS approach may be associated with higher learner satisfaction, improved application of higher-order knowledge, better OSCE-based clinical performance, and higher levels of self-perceived clinical competence, although it was accompanied by an increased instructional workload. Taken together, these results indicate that the CBL-BOPPPS model may represent a feasible and potentially valuable teaching approach for competency-oriented neurosurgical residency education.

Contemporary neurosurgical residency training has increasingly shifted toward competency-based medical education (CBME), which emphasizes observable performance, clinical reasoning, and progressive entrustment rather than solely time-based exposure (3, 4). Within this paradigm, teaching strategies must facilitate the integration of knowledge with decision-making and professional behaviors. The results of the present study appear to be consistent with this educational framework, as the CBL-BOPPPS model was associated with improvements in several learning outcomes, particularly those related to clinical reasoning, treatment planning, and patient communication. Compared with undergraduate learners, residents are required to translate theoretical knowledge into real-time clinical decisions under supervision, which may explain why structured case-based approaches can be particularly relevant in residency-level training (16).

The observed educational benefits likely reflect the complementary mechanisms of CBL and the BOPPPS model. CBL promotes active engagement and contextualized reasoning by anchoring learning in authentic clinical scenarios (5, 8, 16). However, prior studies have highlighted variability in CBL implementation, which may limit consistency across sessions. The BOPPPS framework introduces a standardized instructional sequence—bridging prior knowledge, clarifying objectives, assessing baseline understanding, facilitating participatory learning, and reinforcing outcomes through feedback and summary (9, 10).This structure may enhance instructional coherence while preserving learner-centered engagement. Our findings extend previous reports of BOPPPS effectiveness in postgraduate and residency education (9, 17, 18). to the neurosurgical training context, suggesting that structured instructional design can improve the reproducibility and educational impact of case-based teaching.

Importantly, improvements were observed not only in learner perceptions but also in OSCE-based assessments of clinical behavior. Performance gains were most pronounced in domains requiring synthesis, prioritization, and communication, rather than in isolated interpretive tasks. This pattern aligns with educational research demonstrating that active, feedback-oriented instructional designs have a stronger impact on behavioral change than on knowledge acquisition alone (14). The use of standardized patients and structured assessment rubrics further enhances the validity of these findings by enabling objective evaluation of clinically meaningful competencies. From a CBME perspective, such performance-based assessments are essential for capturing skills that written examinations alone cannot adequately measure (11–13, 19).

Notably, no significant differences were observed between groups in clinical data interpretation, radiological image interpretation, diagnostic accuracy, self-learning skills, or basic knowledge mastery. These neutral findings are informative rather than contradictory. Foundational knowledge and image-based interpretation are traditionally well addressed through didactic instruction and independent study, both of which remain effective methods for transmitting structured theoretical content (20–22). Moreover, neurosurgical residents are highly motivated adult learners who routinely supplement formal teaching with self-directed learning and clinical exposure (23, 24).These compensatory learning behaviors may attenuate between-group differences in areas that rely heavily on repetition and exposure rather than on instructional format.

Additionally, both groups participated in identical ward-based teaching sessions with real patients following theoretical instruction. Authentic clinical exposure plays a critical role in consolidating diagnostic and interpretive skills and may outweigh differences arising from classroom-based pedagogy (25, 26). This shared experiential learning likely contributed to the convergence of imaging-related outcomes between the groups.

As expected, the CBL-BOPPPS approach required more faculty preparation time and was associated with a higher perceived teaching workload. Similar findings have been reported in other postgraduate education studies employing active learning strategies, where the development, refinement, and delivery of case-based or small-group learning activities have been noted to increase instructional time and resource requirements compared with traditional lecture-based methods (27–29). Importantly, teaching sustainability ratings did not differ between groups, and overall teaching quality was rated higher in the CBL-BOPPPS group, suggesting that faculty perceived the additional effort as educationally meaningful. Taken together, these findings indicate that the educational advantages of CBL-BOPPPS are domain-specific rather than universal. While the model is particularly effective for enhancing higher-order competencies aligned with CBME goals, traditional methods remain efficient for foundational knowledge delivery. This supports a blended curricular strategy in neurosurgical residency programs, in which structured case-based instruction is selectively applied to competencies less amenable to passive learning, while lectures continue to serve as effective tools for core knowledge transmission (30, 31).

Despite these encouraging findings, several limitations must be acknowledged. First, this study was conducted at a single training center with a relatively small sample size, which may limit the generalizability of the results. Second, participants were allocated using cluster randomization based on predefined rotation blocks rather than individual randomization. Although this approach minimized contamination between teaching methods in the educational setting, some cluster-related bias cannot be completely excluded. Additionally, although multiple educational outcomes were evaluated, this study primarily assessed short-term learning outcomes rather than long-term behavioral changes or clinical performance in real-world clinical settings. Future multicenter studies with larger sample sizes and extended follow-up periods are necessary to further evaluate the educational impact of the CBL-BOPPPS model in neurosurgical residency training.

## 5 Conclusion

This study suggests that integrating CBL with the BOPPPS instructional model may enhance residents’ learning outcomes in neurosurgical training. Compared to traditional lecture-based teaching, the CBL-BOPPPS approach was associated with improved performance in theoretical knowledge, clinical case analysis, and OSCE assessments. These findings indicate that the CBL-BOPPPS model may promote active learning and foster the development of clinical reasoning skills among neurosurgical residents. Further multicenter studies with larger sample sizes are needed to validate these results.

## Data Availability

The raw data supporting the conclusions of this article will be made available by the authors, without undue reservation.
